# Normalized Multipotential Redox Coding of DNA Bases for Determination of Total Nucleotide Composition

**DOI:** 10.1021/acs.analchem.3c02023

**Published:** 2023-08-14

**Authors:** David Kodr, Mayreli Ortiz, Veronika Sýkorová, Cansu Pinar Yenice, Zbigniew J. Lesnikowski, Ciara K. O’Sullivan, Michal Hocek

**Affiliations:** † Institute of Organic Chemistry and Biochemistry, Czech Academy of Sciences, Flemingovo namesti 2, CZ-16000 Prague 6, Czech Republic; ‡ Departament d’Enginyeria Química, Universitat Rovira i Virgili, 26 Països Catalans, 43007 Tarragona, Spain; § Department of Organic Chemistry, Faculty of Science, Charles University, Hlavova 8, Prague 2 CZ-12843, Czech Republic; ∥ Laboratory of Medicinal Chemistry, Institute of Medical Biology PAS, Lodowa 106, 92-232 Łódź, Poland; ⊥ Institució Catalana de Recerca i Estudis Avançats, Passeig Lluís Companys, 23, 08010 Barcelona, Spain

## Abstract

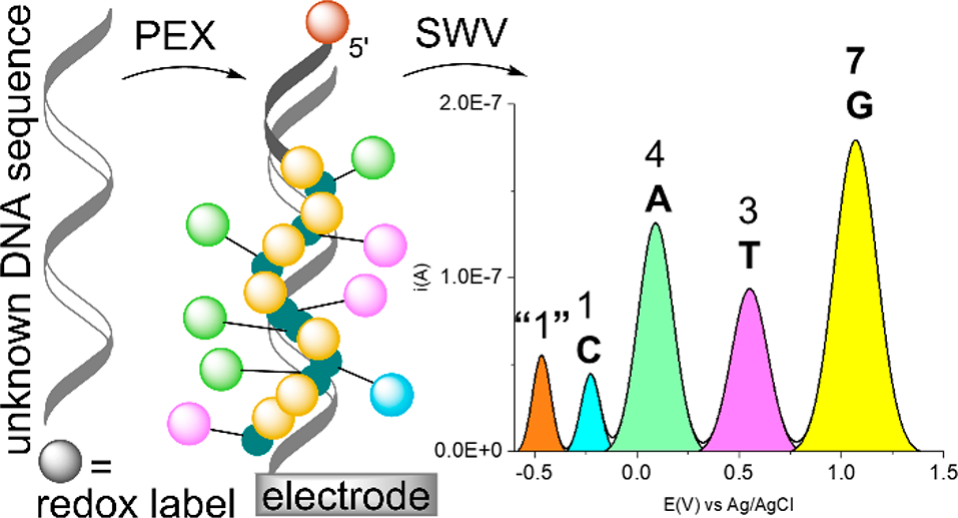

The previously reported approach of orthogonal multipotential redox coding of all four DNA bases allowed only analysis of the relative nucleotide composition of short DNA stretches. Here, we present two methods for normalization of the electrochemical readout to facilitate the determination of the total nucleotide composition. The first method is based on the presence or absence of an internal standard of 7-deaza-2′-deoxyguanosine in a DNA primer. The exact composition of the DNA was elucidated upon two parallel analyses and the subtraction of the electrochemical signal intensities. The second approach took advantage of a 5′-viologen modified primer, with this fifth orthogonal redox label acting as a reference for signal normalization, thus allowing accurate electrochemical sequence analysis in a single read. Both approaches were tested using various sequences, and the voltammetric signals obtained were normalized using either the internal standard or the reference label and demonstrated to be in perfect agreement with the actual nucleotide composition, highlighting the potential for targeted DNA sequence analysis.

The growing demand for rapid, precise, and cost-effective targeted DNA sequence analysis has resulted in the development of a plethora of platforms for the detection of nucleic acids.^1,2^ Electrochemical detection is a particularly attractive alternative to optical-based detection methods, due to its high sensitivity, comparatively simple instrumentation, and relatively low cost.^3,4^ As the reduction and oxidation reactions of nucleobases or their residues require either high negative or high positive potentials, a wide range of diverse labels have been employed for the electrochemical detection of nucleic acids.^5^ Several redox labels, including reducible nitroaryl,^6^ azidoaryl,^7^ or benzofurazane,^8^ as well as oxidizable ferrocene derivatives,^9,10^ methylene blue,^11−17^ Nile blue,^18,19^ or tyrosine,^20,21^ tethered to the nucleobase part of nucleotides have been studied, and some of them have been successfully exploited as labels enzymatically incorporated into DNA for applications in electrochemical (EC) detection and sensing. This gave a rise to an idea of using electrochemical redox labels for multipotential coding of all four nucleobases as an alternative to multicolor-based fluorescence methods,^22,23^ where an immobilized DNA primer hybridizes to the sequence under interrogation, and the primer is then extended via enzyme-mediated primer extension. However, due to quite narrow potential windows (differing on various electrodes) applicable for DNA analysis and difficulties in designing and synthesizing orthogonal and ratiometric redox labels, this has remained elusive for a long time. Only recently, we have developed and reported^24^ a combination of four oxidizable redox labels (dicarba-nido-undecaborate, 3,3-iron-bis(1,2-dicarbollide), ferrocene, and 7-deazaguanine) that can be attached to four nucleotides, enzymatically incorporated by DNA polymerase in oligonucleotide strands containing up to 16 modified nucleotides in a row, and due to their unique distinguishable redox potentials, these labels could simultaneously and ratiometrically detect using SWV on glassy carbon electrodes. This was the first proof-of concept demonstration of the redox coding of DNA bases that allowed the determination of the relative content (ratio) of the four nucleobases in an unknown sequence of DNA, but the quantification of the total number of each nucleobase remained a challenge. In this work, we address this issue, comparing the use of an internal standard in the primer or employing a fifth redox label that facilitates a referential ratiometric analytical signal, allowing the determination of the exact number of each redox label, and thus each nucleobase, present in a specific DNA sequence.

Initially, we designed an approach relying on the use of prelabeled primers with one of the already established redox labels as the internal standard (Method A). Specifically, we employed primers containing one or two 7-deaza-2′-deoxyguanosines (dG*) as the simplest of the four redox labels (Figure 1A). These modified oligonucleotide (ON) primers can be readily synthesized or obtained from commercial ON suppliers. Performing two parallel primer extensions (PEX), one with the nonmodified primer and other with the prelabeled primers containing either one or two dG* nucleotides, and consecutive EC analysis of the studied sequence would result in two voltammograms differing in the intensity of the dG* signal (by “+1” or “+2″, respectively). Subsequent subtraction of the two relative ratios should facilitate determination of the total nucleotide composition of the sequence under study.

**Figure 1:**
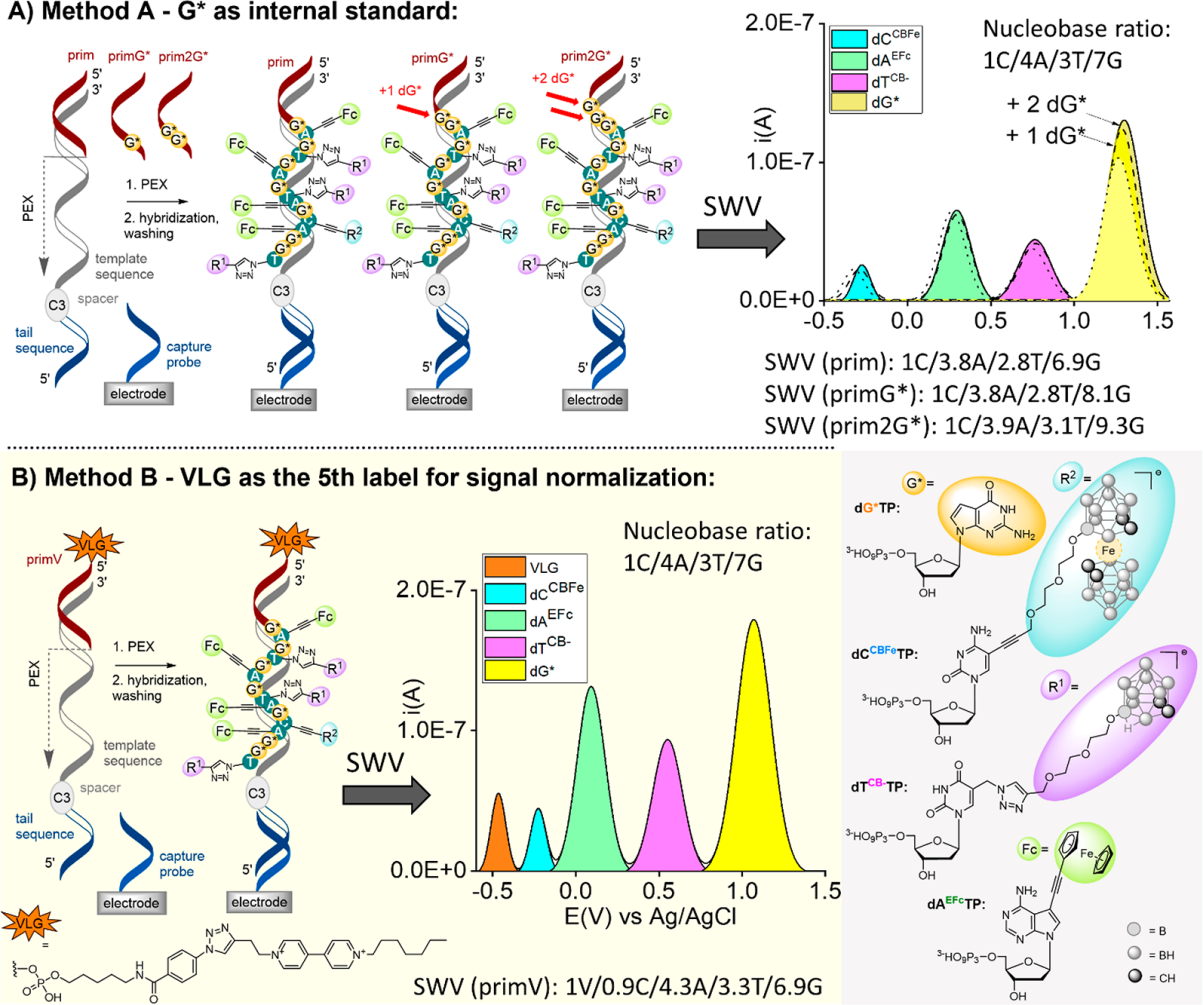
PEX with KOD XL DNA polymerase, four modified nucleoside triphosphates: dG*TP, **dC**^**CBFe**^**TP**, **dT**^**CB-**^**TP**, **dA**^**EFc**^**TP**, and template temp_C3_^1C/4A/3T/7G^. Captured on the electrode and SWV analysis employing (A) the internal standard method using a set of primers: prim, primG*, and prim2G*. Nucleotide composition is calculated upon two consecutive measurements using two different primers and subtraction of dG* signal intensity. (B) Referential reporter method using VLG-modified primer prim**V**. Nucleotide composition is acquired in single measurement as the fifth referential signal is integrated as “1”. SWV values with baseline correction from representative measurement are shown. For primary data, see the SI.

To demonstrate the concept, we carried out PEX reactions with all four modified nucleoside triphosphates (**dN**^**X**^**TPs**: dG*TP, **dC**^**CBFe**^**TP**, **dT**^**CB-**^**TP**, **dA**^**EFc**^**TP**; Figure 1) according to our previously established protocol.^24^ The parallel reactions were set up with a natural primer (prim; for sequences see Table S1) and primer containing either one or two dG* redox labels (primG* and prim2G*, respectively). The template contained 20-mer single stranded ON at its 5′-end tethered via a C3 spacer and complementary to the capture probe immobilized on the electrode surface. The outcome of the PEX reactions carried out with the newly designed primers containing the redox labels was monitored using PAGE analysis with GelRed poststaining and fluorescence visualization (Figures S1 and S2). Square wave voltammetry (SWV) analysis revealed proportional signals in agreement with with the expected ratio(s) of nucleotide composition. More importantly, the signal intensity at +1.1 V vs Ag/AgCl (originating from the dG*) was increased by “+1” or “+2” for the PEX products synthesized using primG*, or prim2G^*^, respectively. The total nucleotide composition was calculated from the two consecutive measurements using two of the three used primers.

To avoid the need for laborious consecutive measurements, we proposed a novel alternative approach exploiting a fifth reference redox label tethered to the 5′-end of the primer (Method B, Figure 1B). This concept is more challenging, as the other four redox labels already occupy a large part of the potential window available while using glassy carbon electrodes. The potential window gap for a fifth EC label was from −0.3 to −0.9 V vs Ag/AgCl. We selected viologen^25^ (**VLG** in Figure 1B), to label the 5′-end of the primer (prim**V**) in the enzymatic PEX reaction, and the formation of the full-length product was confirmed by PAGE and UPLC-MS analysis (Figure S3 and Table S2). In SWV of the PEX product, we observed an additional redox signal at potential −0.4 V well-differentiated from the signals of the other four redox labels. Using this approach, we successfully demonstrated the determination of the total nucleotide composition upon integration of all signals and assignment of a theoretical value of 1 to the signal of viologen, thus elucidating the nucleotide composition in a single read.

Both of these approaches, either using the internal standard primer or the reference redox label, were further tested on two other sequences that differed in the numbers and positions of redox labeled nucleotides (Table 1; Figures S6–S11 in the SI). We obtained ratiometric responses of the signal intensities corresponding well with the theoretical ratio of the nucleobase composition in the newly synthesized DNA sequences in all experiments (each carried out in three replicates).

**Table 1:** SWV Analysis of Nucleobase Composition of PEX Products

Template	Method	temp_C3_^2C/6A/2T/6G^	temp_C3_^2C/2A/2T/7G^
Theoretical ratio		2C/6A/2T/6G	2C/2A/2T/7G
SWV with prim	A	2C/5.8A/2.2T/5.9G	2C/2.1A/2.0T/7.1G
SWV with primG*	A	2C/6.2A/2.2T/6.9G	2C/2.0A/2.4T/8.1G
SWV with prim2G*	A	2C/5.8A/2.3T/7.9G	2C/1.8A/1.7T/9.2G
SWV with prim**V**	B	1V/2.1C/5.8A/2.3T/5.8G	1V/2.1C/2.2A/2.1T/7.2G

In conclusion, we developed two new approaches for normalization of the EC signals in redox coding that facilitate the determination of the exact nucleotide composition of a short DNA sequence. While the internal standard method did not require an additional redox label, two parallel analyses were necessary. On the other hand, the conceptually novel approach using viologen as the fifth redox label tethered to the 5′-end of the primer sequence as a reference for signal normalization is more straightforward and is universally useful for multiple applications in targeted EC sequence analysis and genotyping.

## Supporting Information Available

Full experimental part, methods and procedures, full characterization of all compounds, tables of sequences of oligonucleotides, additional and full uncut gels, and additional electrochemical measurements (PDF)
