# Supplementary material for: Normalized Multipotential Redox Coding of DNA Bases for Determination of Total Nucleotide Composition
Source: Anal Chem. 2023 Aug 14;95(34):12586–9. doi: 10.1021/acs.analchem.3c02023 (PMC10469368; doi:10.1021/acs.analchem.3c02023)
Supplement: Supplementary file 1 — ac3c02023_si_001.pdf [file ac3c02023_si_001.pdf]

Supporting Information

Normalized Multipotential Redox Coding of DNA Bases for Determination of Total Nucleotide Composition

David Kodr,[a+] Mayreli Ortiz,[b+] Veronika Sýkorová,[a] Cansu Pinar Yenice,[b] Zbigniew J. Lesnikowski,[d] Ciara K. O'Sullivan,[b,e]* and Michal Hocek[a,c]*

[a] *Institute of Organic Chemistry and Biochemistry, Czech Academy of Sciences, Flemingovo namesti 2, CZ-16000 Prague 6, Czech Republic;*

[b] *Departament d'Enginyeria Química, Universitat Rovira i Virgili, 26 Països Catalans, 43007, Tarragona, Spain;*

[c] *Department of Organic Chemistry, Faculty of Science, Charles University in Prague, Hlavova 8, Prague 2, CZ-12843, Czech Republic.*

[d] *Laboratory of Medicinal Chemistry, Institute of Medical Biology PAS, Lodowa 106, 92-232 Łódź, Poland*

[e] *Institució Catalana de Recerca i Estudis Avançats, Passeig Lluís Companys, 23, 08010, Barcelona, Spain*

**ciara.osullivan@urv.cat*

**hocek@uochb.cas.cz*

+ these authors contributed equally

Contents

1. Experimental section S2
2. Electrochemical analyses S7
3. References S17

Experimental section

General remarks

All gels were analysed by fluorescence imaging using Typhoon FLA 9500 (GE Healthcare). UV-Vis spectra were measured at room temperature in a NanoDrop1000 (ThermoScientific). Synthetic oligonucleotides (for sequences see Table S1) were purchased from biomers.net GmbH (Germany), except the 7-deazaG containing primers which were purchased from metabion GmbH. The MS spectra were measured on UPLC Agilent 1290 Infinity II bio system equipped with DAD and LC/MSD XT MS (operating in negative mode) detectors, with bioZen™ Oligo LC column (1.7 µm, 50×2.1 mm; Phenomenex®) using a linear gradient of 12 mM Et_3N and 300 mM HFIP in H_2O to 12 mM Et_3N and 300 mM HFIP in MeOH in 10 min. Mass spectra were deconvoluted using OpenLAB CDS CS LC/MS Deconvolution Bioanalysis software. Natural nucleoside triphosphates (dATP, dGTP, dTTP, dCTP) were purchased from ThermoScientific. Modified nucleoside triphosphates were prepared according to the published protocols.[1, 2] KOD XL DNA polymerase and corresponding polymerase reaction buffer from Merck (Sigma Aldrich). Milli-Q water was used for all experiments. Native loading dye used after PEX reactions contains: 0.07% [w/v] bromophenol blue, 0.07% [w/v] xylene cyanol and 13.3% [w/v] saccharose. Samples after PEX reactions were analysed by a 12.5% native PAGE (acrylamide/bisacrylamide 19:1), (4 h, 8 °C, 1× TBE). Other chemicals were of analytical grade. Samples after PCR reactions were separated with a 2% agarose gel (Agarose, research grade, Serva) in 0.5× TBE buffer.

All reagents used for electrochemical analysis were analytical grade and used as received. Ultrapure water (18 MΩ cm) purified by a Simplicity Water Purification System (Millipore, France) was used for preparing the electrochemical solutions, which were deoxygenated by bubbling nitrogen gas for 20 minutes immediately before measurement. Strontium nitrate ($Sr(NO_3)_2$), potassium hydroxide (KOH), sodium phosphate (PBS) and TWEEN® 20 were purchased from Thermo Fisher Scientifics (Spain). Hydrochloric acid (35% v/v) was received from Panreac (Spain), while N-(4-aminophenyl)-maleimide, sodium nitrite ($NaNO_2$), potassium ferricyanide ($K_3[Fe(CN)_6]$), potassium chloride (KCl) and Sigma H7140 hybridization solution were acquired from Merck (Sigma Aldrich) (Spain). The glassy carbon bars were purchased from IJ Cambria

Scientific Ltd, UK. and the teflon covered glassy carbon electrodes were produced in facilities of Universitat Rovira i Virgili.

All electrochemical measurements, were performed with a potentiostat/galvanostat PGSTAT 12 Autolab controlled with Nova 2.1.4 software. The raw data was baseline corrected using the polynomial algorithm provided in the Nova 2.1.4 software.

Table S1 List of sequences of primers and templates

Name	Size (nt)	Sequence (5' → 3')
prim	16	5'-CATGGGCGGCCATTGG-3'
primG*	16	5'-CATGGGCGGCCATTG[7-deazaG]-3'
Prim2G*	16	5'-CATGGGCGGCCATT[7-deazaG][7-deazaG]-3'
primV	16	5'-Viologen-CATGGGCGGCCATTGG-3'
temp$_{C3}^{2C/6A/2T/6G}$	53	5'-ATTACGACGAACTCAATGA-**C3**-ACTCTGCTAGCTCTCTCCAATGGCCGCCCATG-3'
temp$_{C3}^{2C/2A/2T/7G}$	50	5'-ATTACGACGAACTCAATGAA-**C3**-CCTACGCACGCCTCCAATGGCCGCCCATG-3'
temp$_{C3}^{1C/4A/3T/7G}$	52	5'-ATTACGACGAACTCAATGAA-**C3**-ACCTGCTACTCACTCCCAATGGCCGCCCATG-3'
Capture probe	35	5'-TTCATTGAGTTCGTCGTAATTTTTTTTTTTTTTTT-3'-**C6**-**SH**

C3 = –$(CH_2)_3$– spacer; **C6-SH** = –$(CH_2)_6$–SH cap; in the template ONs the segments forming a duplex with the primer are underlined; the complementary strands forming primer (orange) and capture probe (blue) duplexes are designated by the respective colors (orange and blue).

1.1 Primer extension

1.1.1 Analytical PEX and sample for electrochemical analysis

The reaction mixture (20 µL) contained template $temp_{C3}^{2C/6A/2T/6G}$, or $temp_{C3}^{2C/2A/2T/7G}$, or $temp_{C3}^{1C/4A/3U/7G}$ (2 µM, 2 µL), primer prim, or primG*, or prim2G*, or primV (2 µM, 2 µL), modified **dN^{X}TPs** (2 mM, 2 µL), KOD XL DNA polymerase (1.2 U) and reaction buffer (10×, 2 µL) as supplied by the manufacturer. The reaction mixture was incubated for 30 minutes at 60 °C. The PEX reaction was stopped by cooling to 8 °C. For PAGE analysis was the PEX sample mixed with a native loading dye upon running stained with GelRed® and visualised using fluorescence imaging (Figure S1-S3).

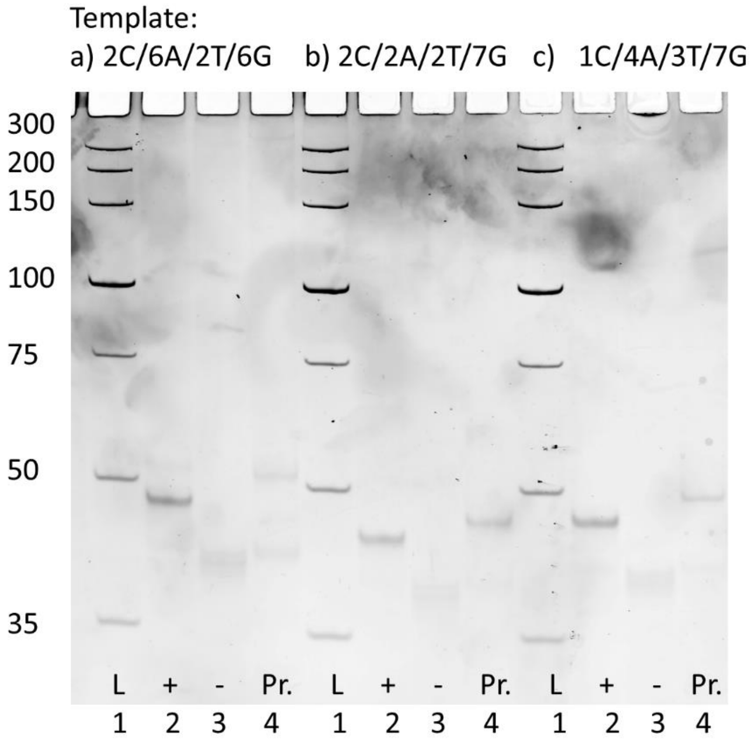

Figure S1 Native PAGE analysis of PEX with KOD XL DNA polymerase using four modified dNTPs, primer primG* and a) $temp_{C3}^{2C/6A/2T/6G}$; b) $temp_{C3}^{2C/2A/2T/7G}$; c) $temp_{C3}^{1C/4A/3T/7G}$; (1) dsDNA ladder with denoted length; (2) positive control: natural dNTPs; (3) negative control: without studied dNTPs; (4) reactions containing **dA^{EFc}TP**, **dC^{CBFe}TP**, **dT^{CB-}TP**, **dG*TP**.

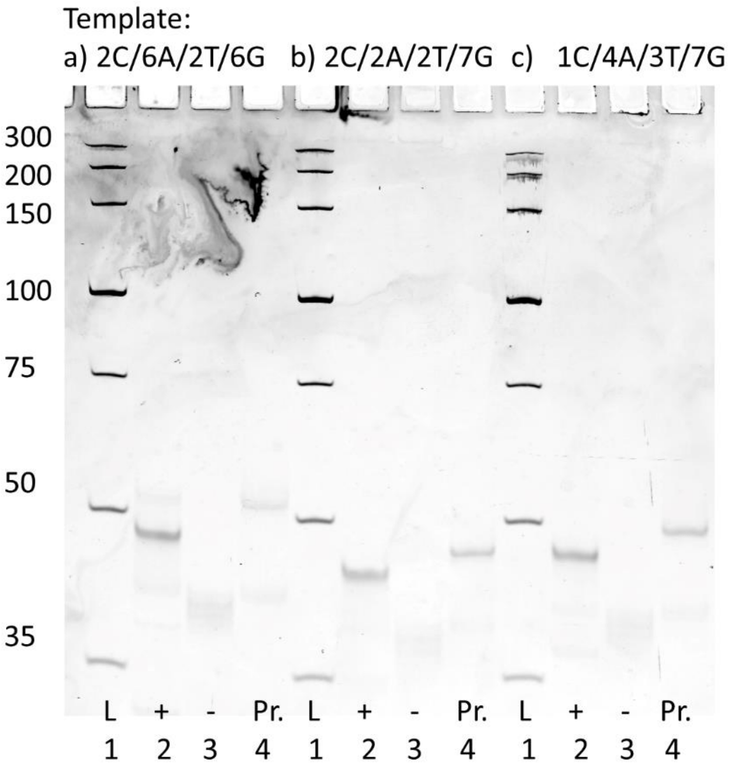

Figure S2 Native PAGE analysis of PEX with KOD XL DNA polymerase using four modified dNTPs, primer prim2G* and a) temp$_{C3}$$^{2C/6A/2T/6G}$; b) temp$_{C3}$$^{2C/2A/2T/7G}$; c) temp$_{C3}$$^{1C/4A/3T/7G}$; (1) dsDNA ladder with denoted length; (2) positive control: natural dNTPs; (3) negative control: without studied dNTPs; (4) reactions containing **dA^{EFc}TP**, **dC^{CBFe}TP**, **dT^{CB-}TP**, **dG*TP**.

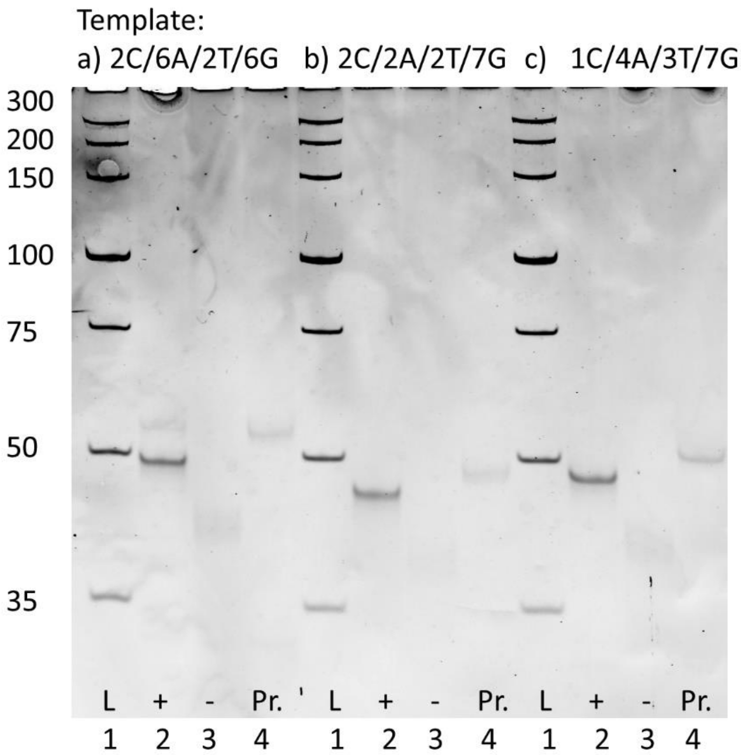

Figure S3 Native PAGE analysis of PEX with KOD XL DNA polymerase using four modified dNTPs, primer primV and a) $temp_{C3}^{2C/6A/2T/6G}$; b) $temp_{C3}^{2C/2A/2T/7G}$; c) $temp_{C3}^{1C/4A/3T/7G}$; (1) dsDNA ladder with denoted length; (2) positive control: natural dNTPs; (3) negative control: without studied dNTPs; (4) reactions containing **dA^{EFc}TP**, **dC^{CBFe}TP**, **dT^{CB-}TP**, **dG*TP**.

1.1.2 PEX sample for MS analysis

The reaction mixture (50 µL) contained template $temp_{C3}^{2C/6A/2/6G}$, or $temp_{C3}^{2C/2A/2T/7G}$, or $temp_{C3}^{1C/4A/3T/7G}$ (100 µM, 3.2 µL), 5'-Viologen labelled primV (100 µM, 3.2 µL), modified **dN^{X}TPs** (each 4 mM, 2.5 µL), and KOD XL DNA polymerase (3.75 U) in the enzyme reaction buffer (10×, 5 µL) as supplied by the manufacturer. The reaction mixture was incubated for 2 hours at 60 °C. The reaction was stopped by cooling to 8 °C. PEX product was purified using QIA quick® dNTP removal kit.

Table S2 Results of MS analysis of 5'-Viologen labelled fully modified oligonucleotide

oligonucleotide	**M (calcd.) [Da]**	**M (found) [Da]**
**32DNA_2C^{CBFe}_6A^{EFc}_2T^{CB-}_6G***	13401	13393
**29DNA_2C^{CBFe}_2A^{EFc}_2T^{CB-}_7G***	11647	11642
**31DNA_1C^{CBFe}_4A^{EFc}_3T^{CB-}_7G***	12557	12548

1.2 Electrochemical analysis using immobilized capture probe on glassy carbon electrode

1.2.1 Glassy carbon electrode functionalization

Glassy carbon electrodes (GCE) were polished consecutively with sandpaper 600- and 0.3-micron alumina slurries for 10 min. After each polishing, electrode surfaces were rinsed with water in an ultrasonic bath for 5 minutes. Subsequently, the electrodes were electrochemically cleaned using cyclic voltammetry with a scan rate of 100 mV/s, from 0 to -1.2 V vs Ag/AgCl, for 30 scans in a 0.1 M KOH solution. After cleaning, electrode surface was again rinsed with water in an ultrasonic bath for 5 minutes and, dried with N_2. Electrode surface cleanliness were controlled by using square wave voltammetry (SWV) in 0.01 M sodium phosphate + 0.1 M $Sr(NO_3)_2$ (pH 7.4), from -0.6 to +1.6 V vs Ag/AgCl.

1.2.2 Calculation of electroactive area of electrodes

The electrochemical area of each electrode was calculated using the abbreviated Randles–Sevcik equation, where the constant terms can be considered as a single constant when the cyclic voltammograms at different scan rates are recorded at 25 °C (298 K).[3]

$$i_p = 2.69 \times 10^5\, A D^{1/2} n^{3/2} C\, v^{1/2} \qquad \text{(S1)}$$

A = electroactive area of electrode (in cm^2)
D = diffusion coefficient (0.726×10^{-5} cm^2/s) for $K_3[Fe(CN)_6]$ in 0.1 M KCl at 25°C)[4]
n = number of electrons involved in the redox process (1)
C = concentration of the electroactive species in mol/cm^3 (1.0×10^{-6} mol/cm^3)
i_p= peak current intensity in A
v = scan rate in V/s

By plotting i_p vs $v^{1/2}$, the equation corresponds to a linear regression (y = bx) and the electroactive area can be calculated from the slope using the equation:

$$A = slope/(2.69 \times 10^{-5}\ D^{1/2}\ n^{3/2}\ C) \qquad \text{(S2)}$$

In the example below (electrode E1 in table S3):

$$A = slope/(2.69 \times 10^{5}(0.726 \times 10^{-5})^{1/2}\ (1x10^{-6})) \qquad \text{(S3)}$$

$$A= 4.74 \times 10^{-5}/7.24\ x10^{-4}= 0.65 \times 10^{-1} = 0.0654\ cm^2 \qquad \text{(S4)}$$

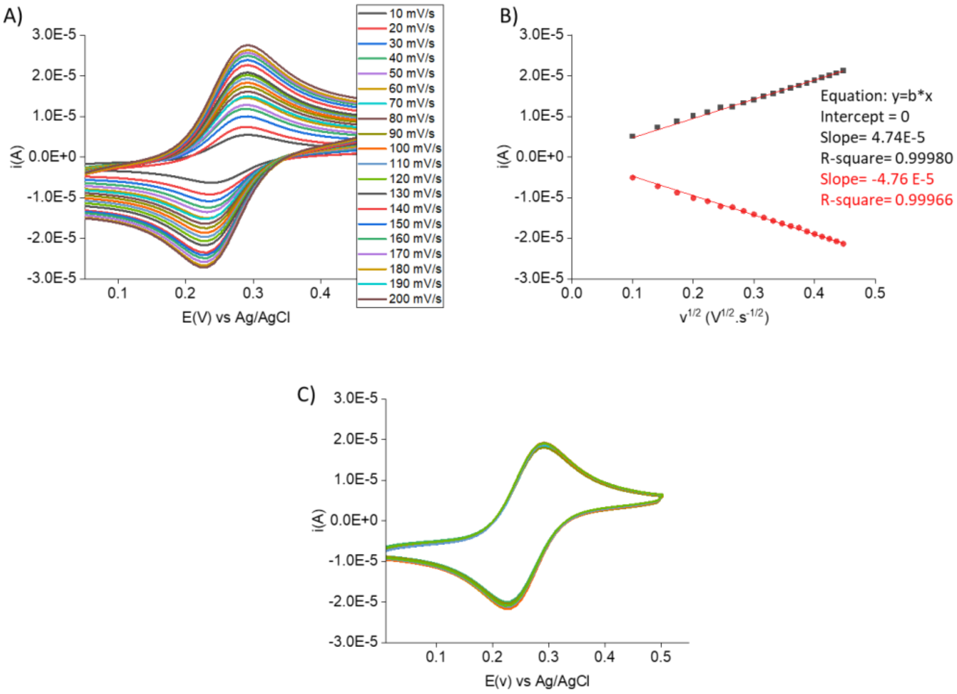

Figure S4 Example of the determination of the electroactive area of an electrode. A) Different voltammograms recorded in a 1 mM $K_3[Fe(CN)_6]$ + 0.1 M KCl solution at different scan rates. B) Current intensity of anodic and cathodic peak of each voltammogram vs the square root of the corresponding scan rate and the linear regression. C) The cyclic voltammograms of the electrodes used for the experiments overlapped to demonstrate their similar performance characteristics, achieved with the cleaning procedure described above.

Table S3. Electroactive areas of the used electrodes

electrode	Electroactive area (cm^2)
**E1**	0.0654
**E2**	0.0645
**E3**	0.0671
**E4**	0.0648
**E5**	0.0641
**E6**	0.0666
**E7**	0.0677
**E8**	0.0638
**E9**	0.0680
**E10**	0.0670
**E11**	0.0666
**E12**	0.0675

The average surface area was A = (0.066 ± 0.002) cm^2 (2.3% Relative Standard Deviation)

1.2.3 Electrochemical Grafting of Diazonium Salts on GCE

Firstly, 2 mL of 10 mM $NaNO_2$ water solution were added to a 2 mL ice-cold solution of 10 mM N-(4-aminophenyl)-maleimide in 0.5 M HCl and stirred for 10 min. Finally, the carbon electrode was immersed in the solution together with the platinum counter electrode and the reference Ag/AgCl electrode and the resulted diazonium salt (Figure S5) was electrochemically grafted on carbon electrode surface as described previously[3] with the potential cycled between +0.1 and -0.6 V vs Ag/AgCl for 5 cycles. After grafting, the electrode surfaces were rinsed with water in an ultrasonic bath for 1 minute and, dried with N_2. Another SWV measurement was carried out to control the surface grafting.

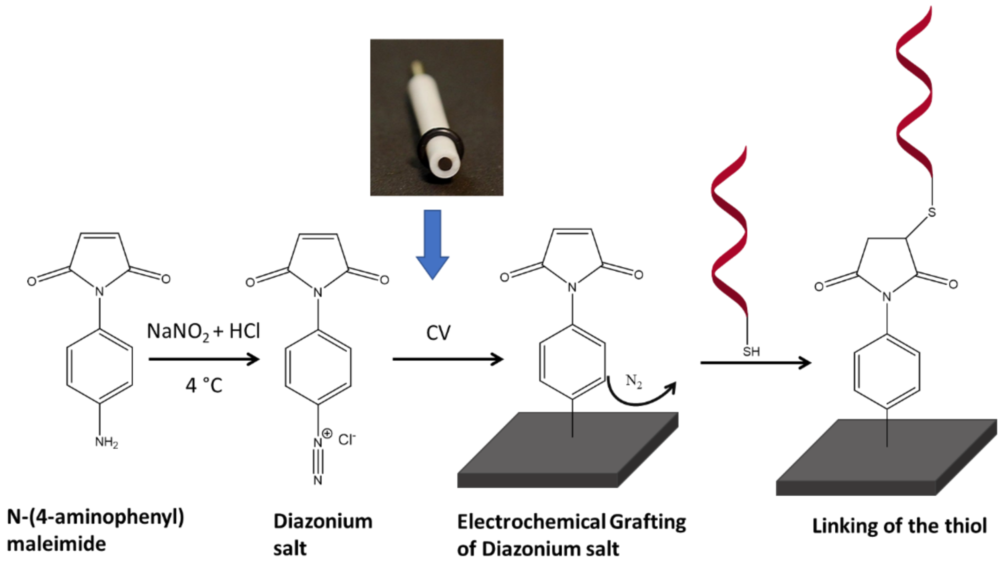

Figure S5 Schematic representation of the electrochemical grafting of the diazonium salt onto a glassy carbon electrode and subsequent immobilization of a thiolated DNA probe by covalent binding to the maleimide group. Actual picture of the glassy carbon electrode in the inset.

1.2.4 Immobilization of capture probe

Blue tips (capacity of 100-1000 µL) were cut and adjusted to create chambers on electrodes. A 5 µM capture probe in 0.01 M PBS (pH 7.4) was drop casted on. The chambers were covered with parafilm and incubated for 16 hours at 4 °C. Subsequently, the electrodes were rinsed with 0.01 M PBS (pH 7.4) + 0.05% TWEEN® 20 and dried with N_2. Then the electrodes were incubated with a freshly prepared 100 µM of mercaptohexanol solution for 30 minutes at 22 °C, to inactivate the remaining maleimide groups of the surface. Electrodes were rinsed first with deionized water and then with 0.01 M PBS (pH 7.4) under stirring for 15 minutes and dried with N_2.

1.2.5 Primer extension and hybridization on the glassy carbon electrode

PEX was performed as described above. New chambers were created on the electrode for hybridization of the target. A hybridization solution was prepared to contain 5 µL of PEX product, 5 µL of 0.02 M PBS (pH 7.4) (2x) and 2 µL of hybridization Sigma H7140 (2×) and drop casted on electrodes for 60 minutes incubation at 37 °C. The electrode were removed from chambers, washed with 0.01 M PBSv(pH 7.4) + 0.05% TWEEN® 20 and immersed in 10 mL of same solution and stirred for 15 minutes to remove any non-hybridized PEX product. Finally, the electrodes

were washed again with 0.01 M PBS (pH 7.4) and the electrochemical measurement was immediately carried out.

1.2.6 Electrochemical measurements on GCE using optimised parameters

Measurements were carried out with an external Ag/AgCl (1 M KCl) reference electrode, a gold wire as a counter and glassy carbon as a working electrode. Electrochemical measurements were carried out by SWV in 0.01 M PBS (pH 7.4) + 0.1 M $Sr(NO_3)_2$, from -0.6 to + 1.5 V vs Ag/AgCl using a 1 mV step, 20 mV modulation amplitude and 10 Hz of frequency.

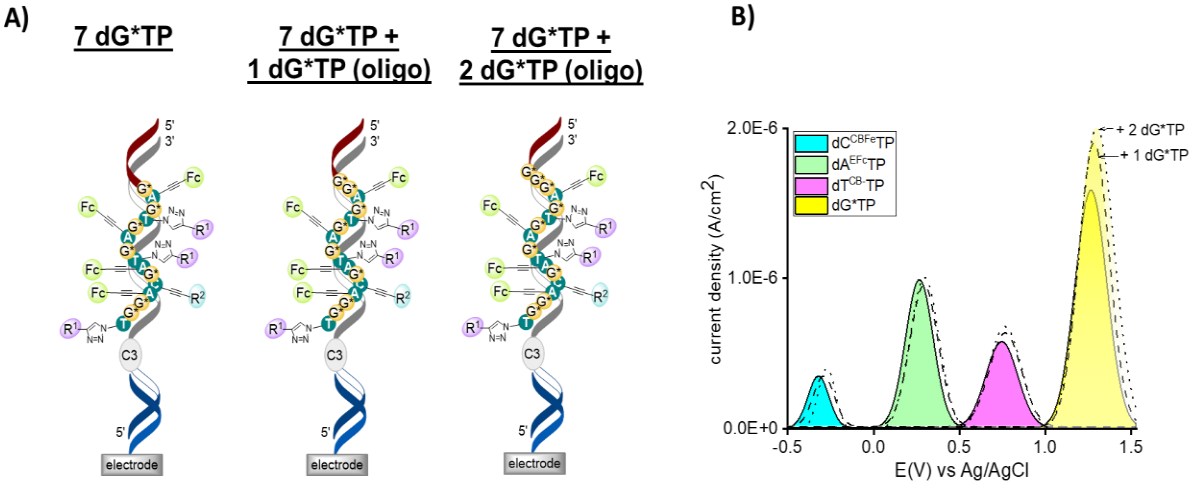

	7 dG*TP	7 dG*TP + 1 dG*TP (oligo)	7 dG*TP + 2 dG*TP (oligo)
Theoretical Ratio	1C/4A/3T/7G	1C/4A/3T/8G	1C/4A/3T/9G
Experimental ratio	1C/3.8A/2.6T/6.9G	1C/3.8A/2.8T/8.1G	1C/3.9A/3.1T/9.3G

Figure S6 Experimental data (SWV) for reference readout using 7-deazaG signal subtraction (theoretical ratio: 1C/4A/3T/7G; 1C/4A/3T/7G + 1G* and 1C/4A/3T/7G + 2G*). A) Schematic representation of PEX products (containing none, one or two 7-deazaG on the oligo for further quantification of the labelled bases) and hybridized on a capture probe immobilized on a glassy carbon electrode. B) Raw data of the SWV recorded for each PEX product that was sequentially hybridized on the same electrode. The voltammograms (in A) were divided by the electroactive area of the electrode (in cm^2) to provide the current density (A/cm^2) in Y-axis. The SWVs were overlapped for better visualization, and the calculation of the experimental ratio of the labelled bases by the integration of the area of SWV peaks.

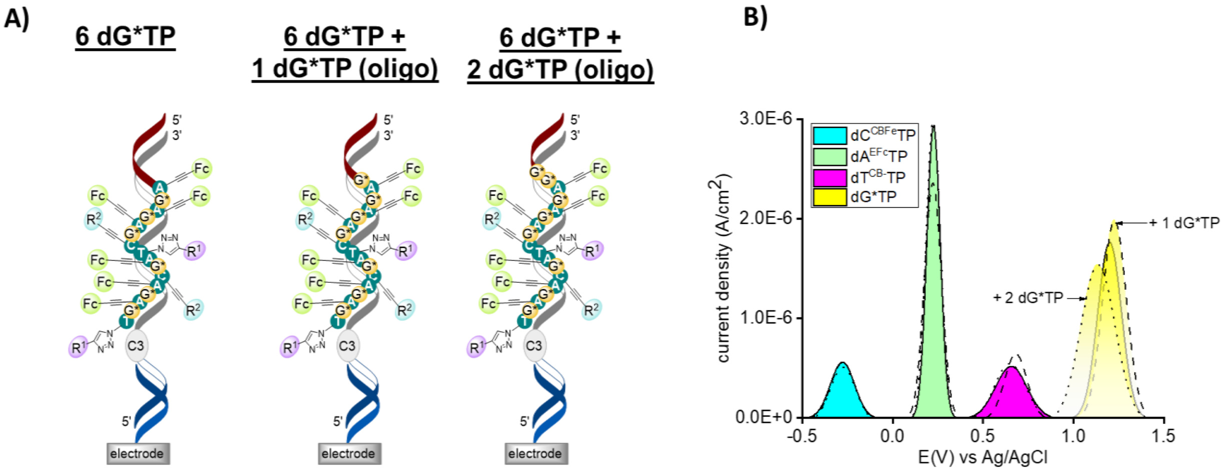

	6 dG*TP	6 dG*TP + 1 dG*TP (oligo)	6 dG*TP + 2 dG*TP (oligo)
Theoretical Ratio	2C/6A/2T/6G	2C/6A/2T/7G	2C/6A/2T/8G
Experimental ratio	2C/5.8A/2.3T/5.9G	2C/6.2A/2.2T/6.9G	2C/5.8A/2.3T/7.9G

Figure S7 Experimental data (SWV) for reference readout using 7-deazaG signal subtraction (theoretical ratio: 2C/6A/2T/6G; 2C/6A/2T/6G + 1G* and 2C/6A/2T/6G + 2G*). A) Schematic representation of PEX products (containing none, one or two 7-deazaG on the oligo for further quantification of the labelled bases) and hybridized on a capture probe immobilized on a glassy carbon electrode. B) Raw data of the SWV recorded for each PEX product that was sequentially hybridized on the same electrode. The voltammograms (in A) were divided by the electroactive area of the electrode (in cm^2) to provide the current density (A/cm^2) in Y-axis. The SWVs were overlapped for better visualization, and the calculation of the experimental ratio of the labelled bases by the integration of the area of SWV peaks.

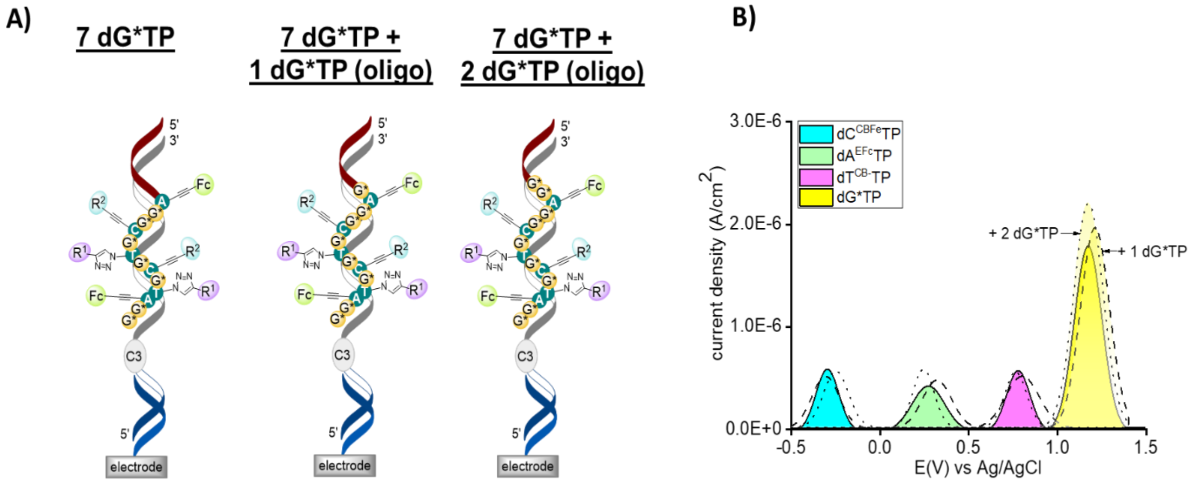

	7 dG*TP	7 dG*TP + 1 dG*TP (oligo)	7dG*TP + 2 dG*TP (oligo)
Theoretical Ratio	2C/2A/2T/7G	2C/2A/2T/8G	2C/2A/2T/9G
Experimental ratio	2C/2.1A/2T/7.1G	2C/2A/2.4T/8.1G	2C/1.8A/1.7T/9.2G

Figure S8 Experimental data (SWV) for reference readout using 7-deazaG signal subtraction (theoretical ratio: 2C/2A/2T/7G; 2C/2A/2T/7G + 1G* and 2C/2A/2T/7G + 2G*). A) Schematic representation of PEX products (containing none, one or two 7-deazaG on the oligo for further quantification of the labelled bases) and hybridized on a capture probe immobilized on a glassy carbon electrode. B) Raw data of the SWV recorded for each PEX product that was sequentially hybridized on the same electrode. The voltammograms (in A) were divided by the electroactive area of the electrode (in cm^2) to provide the current density (A/cm^2) in Y-axis. The SWVs were overlapped for better visualization, and the calculation of the experimental ratio of the labelled bases by the integration of the area of SWV peaks.

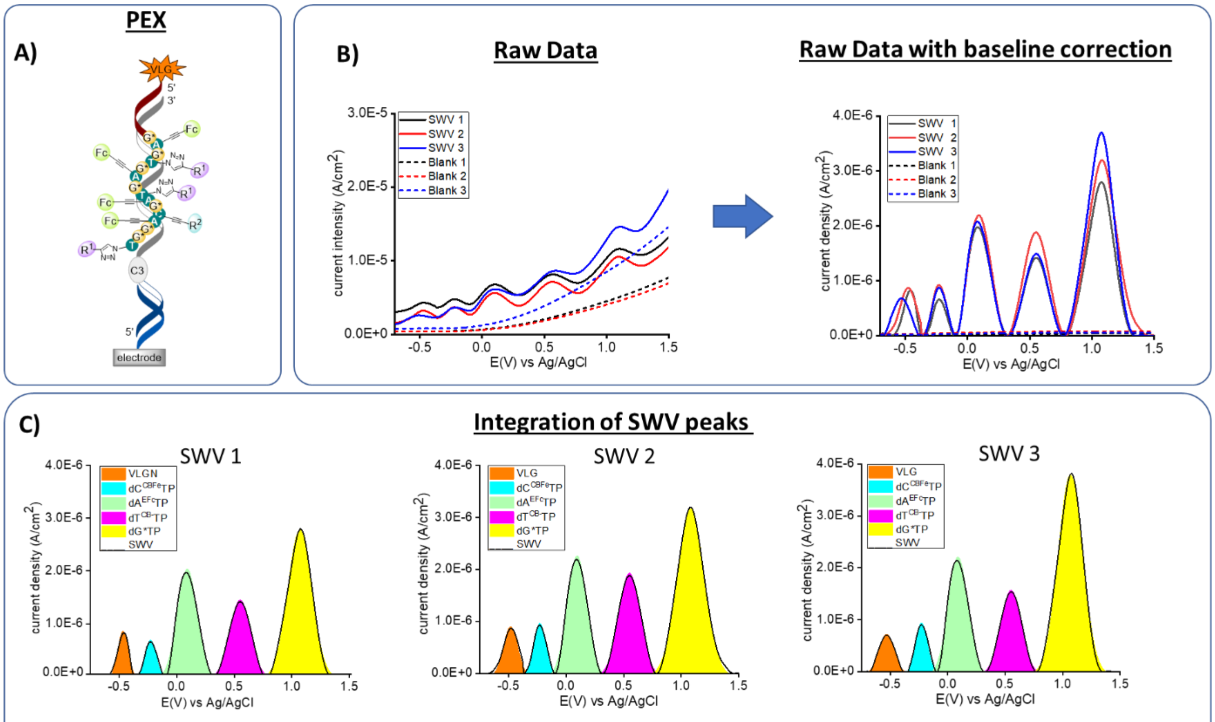

SWV replicate	VLG	dC^{CBFe}TP	dA^{EFe}TP	dT^{CB}-TP	dG*TP
SWV 1	1	0.95	4.13	2.93	6.86
SWV 2	1	0.93	3.60	3.21	6.78
SWV 3	1	0.97	4.01	2.82	7.11
Media	1	0.95	3.91	2.98	6.90
STD		0.02	0.28	0.20	0.17

Figure S9 Experimental data (SWV) for reference labelling using 5'-Viologen primer (theoretical ratio: 1VLG/1C/4A/3T/7G). A) Schematic representation of PEX product hybridized on a capture probe immobilized on a glassy carbon electrode. B) Raw data of three replicates of the SWV recorded for the PEX product in individual electrodes, before and after baseline correction. The voltammograms (in A) were divided by the electroactive area of each electrode (in cm^2) to provide the current density (A/cm^2) in Y-axis. The blank measurement for each electrode were included following a color code to clearly show the difference between the raw data and the noise level. C) Calculation of the experimental ratio of the labelled bases by the integration of the area of SWV peaks of the redox labels taking the viologen as a reference.

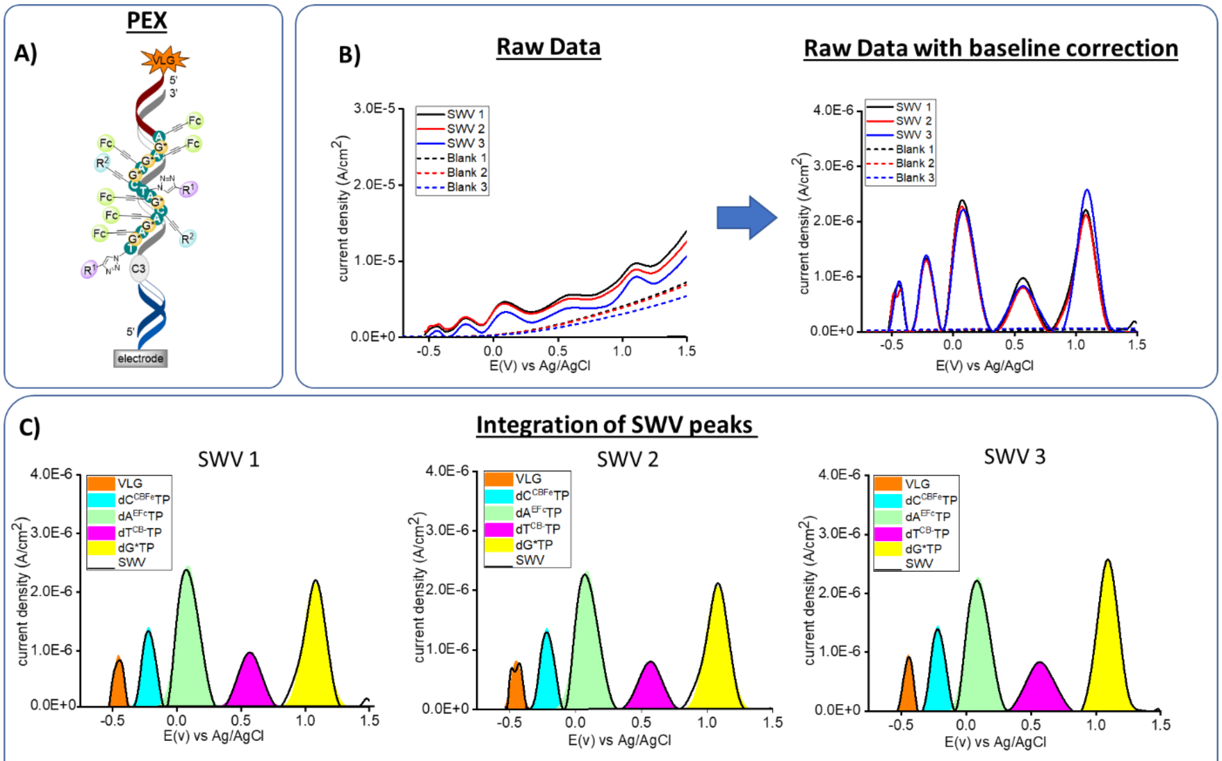

SWV replicate	VLG	$dC^{CBFe}TP$	$dA^{EFe}TP$	$dT^{CB-}TP$	dG*TP
SWV 1	1	2.1	6.2	2.1	5.9
SWV 2	1	2.0	5.8	2.2	5.5
SWV 3	1	2.1	5.5	2.5	5.9
Media	1	2.1	5.8	2.3	5.8
STD	-	0.06	0.35	0.21	0.23

Figure S10 Experimental data (SWV) for reference labelling using 5’-Viologen primer (theoretical ratio: 1VLG/2C/6A/2T/6G). A) Schematic representation of PEX product hybridized on a capture probe immobilized on a glassy carbon electrode. B) Raw data of three replicates of the SWV recorded for the PEX product in individual electrodes, before and after baseline correction. The voltammograms (in A) were divided by the electroactive area of each electrode (in cm^2) to provide the current density (A/cm^2) in Y-axis. The blank measurement for each electrode were included following a color code to clearly show the difference between the raw data and the noise level. C) Calculation of the experimental ratio of the labelled bases by the integration of the area of SWV peaks of the redox labels taking the viologen as a reference. C) Calculation of the experimental ratio of the labelled bases by the integration of the area of SWV peaks of the redox labels taking the viologen as a reference.

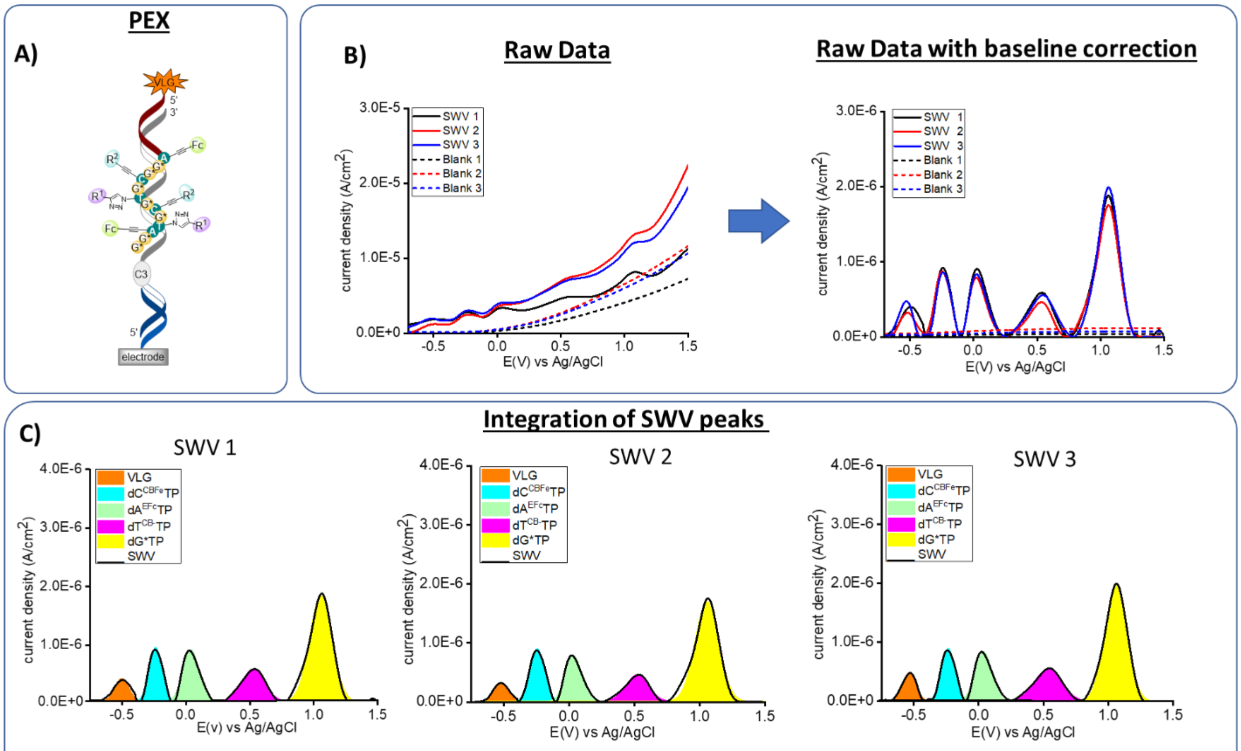

SWV replicate	VLG	dC^{CBFe}TP	dA^{EFe}TP	dT^{CB-}TP	dG*TP
SWV 1	1	2.2	2.5	2.2	7.3
SWV 2	1	2.3	2.1	2.2	7.5
SWV 3	1	1.8	2.0	1.9	6.7
Media	1	2.1	2.2	2.1	7.2
STD		0.81	0.26	0.17	0.42

Figure S11 Experimental data (SWV) for reference labelling using 5'-Viologen primer (theoretical ratio: 1VLG/2C/2A/2T/7G). A) Schematic representation of PEX product hybridized on a capture probe immobilized on a glassy carbon electrode. B) Raw data of three replicates of the SWV recorded for the PEX product in individual electrodes, before and after baseline correction. The voltammograms (in A) were divided by the electroactive area of each electrode (in cm^2) to provide the current density (A/cm^2) in Y-axis. The blank measurement for each electrode were included following a color code to clearly show the difference between the raw data and the noise level. C) Calculation of the experimental ratio of the labelled bases by the integration of the area of SWV peaks of the redox labels taking the viologen as a reference. C) Calculation of the experimental ratio of the labelled bases by the integration of the area of SWV peaks of the redox labels taking the viologen as a reference.

References

1. Kodr, D.; Yenice, C. P.; Simonova, A.; Saftić, D. P.; Pohl, R.; Sýkorová, V.; Ortiz, M.; Havran, L.; Fojta, M.; Lesnikowski, Z. J.; O'Sullivan, C. K.; Hocek, M. Carborane- or Metallacarborane-Linked Nucleotides for Redox Labeling. Orthogonal Multipotential Coding of All Four DNA Bases for Electrochemical Analysis and Sequencing. *J. Am. Chem. Soc.* **2021**, *143*, 7124–7134.

2. Brázdilová, P.; Vrábel, M.; Pohl, R.; Pivonková, H.; Havran, L.; Hocek, M.; Fojta, M. Ferrocenylethynyl Derivatives of Nucleoside Triphosphates: Synthesis, Incorporation, Electrochemistry, and Bioanalytical Applications. *Chem. Eur. J.* **2007**, 13, 9527–9533.

3. Wang, J. Analytical Electrochemistry, 3rd ed.; Wiley-VCH: 876 Hoboken, NJ, 2006.

4. Konopka, S. J.; McDuffie, B. Diffusion Coefficients of Ferri-and Ferrocyanide Ions in Aqueous Media, Using Twin-Electrode Thin-Layer Electrochemistry. *Anal. Chem.* **1970**, 42, 1741-1746.

5. Bartolome, J. P.; Echegoyen, L.; Fragoso A. Reactive Carbon Nano-Onion Modified Glassy Carbon Surfaces as DNA Sensors for Human Papillomavirus Oncogene Detection with Enhanced Sensitivity. *Anal. Chem.* **2015**, 87, 6744-6751.
